# *Toxoplasma gondii-*skeletal muscle cells interaction increases lipid droplet biogenesis and positively modulates the production of IL-12, IFN-g and PGE_2_

**DOI:** 10.1186/1756-3305-7-47

**Published:** 2014-01-23

**Authors:** Alessandra F Gomes, Kelly G Magalhães, Renata M Rodrigues, Laís de Carvalho, Raphael Molinaro, Patrícia T Bozza, Helene S Barbosa

**Affiliations:** 1 Laboratório de Biologia Estrutural, Instituto Oswaldo Cruz, Fundação Oswaldo Cruz, Rio de Janeiro, Brazil; 2 Laboratório de Imunologia e Inflamação, Universidade de Brasília, Brasília, Brazil; 3 Laboratório Cultura de Células, Depto. Histologia e Embriologia, Instituto de Biologia, Universidade do Estado do Rio de Janeiro, Rio de Janeiro, Brazil; 4 Laboratório de Imunofarmacologia, Instituto Oswaldo Cruz, Fundação Oswaldo Cruz, Rio de Janeiro, Brazil

**Keywords:** *Toxoplasma gondii*, Lipid droplets, Skeletal muscle cells, Prostaglandin-E_2_, Cytokines

## Abstract

**Background:**

The interest in the mechanisms involved in *Toxoplasma gondii* lipid acquisition has steadily increased during the past few decades, but it remains not completely understood. Here, we investigated the biogenesis and the fate of lipid droplets (LD) of skeletal muscle cells (SkMC) during their interaction with *T. gondii* by confocal and electron microscopy. We also evaluated whether infected SkMC modulates the production of prostaglandin E_2_ (PGE_2_), cytokines interleukin-12 (IL-12) and interferon-gamma (INF-g), and also the cyclooxygenase-2 (COX-2) gene induction.

**Methods:**

Primary culture of skeletal muscle cells were infected with tachyzoites of *T. gondii* and analysed by confocal microscopy for observation of LD. Ultrastructural cytochemistry was also used for lipid and sarcoplasmatic reticulum (SR) detection. Dosage of cytokines (IL-12 and INF-g) by ELISA technique and enzyme-linked immunoassay (EIA) for PGE_2_ measurement were employed. The COX-2 gene expression analysis was performed by real time reverse transcriptase polymerase chain reaction (qRT-PCR).

**Results:**

We demonstrated that *T. gondii* infection of SkMC leads to increase in LD number and area in a time course dependent manner. Moreover, the ultrastructural analysis demonstrated that SR and LD are in direct contact with parasitophorous vacuole membrane (PVM), within the vacuolar matrix, around it and interacting directly with the membrane of parasite, indicating that LD are recruited and deliver their content inside the parasitophorous vacuole (PV) in *T. gondii*-infected SkMC. We also observed a positive modulation of the production of IL-12 and IFN-g, increase of COX-2 mRNA levels in the first hour of *T. gondii*-SkMC interaction and an increase of prostaglandin E_2_ (PGE_2_) synthesis from 6 h up to 48 h of infection.

**Conclusions:**

Taken together, the close association between SR and LD with PV could represent a source of lipids as well as other nutrients for the parasite survival, and together with the increased levels of IL-12, INF-g and inflammatory indicators PGE_2_ and COX-2 might contribute to the establishment and maintenance of chronic phase of the *T. gondii* infection in muscle cell.

## Background

*T. gondii* is an obligatory intracellular protozoan parasite that resides within a PV, which fails to fuse with host organelles from the endocytic pathway [1,2]. This condition potentially deprives parasites of a large source of nutrients from the host endocytic and exocytic system [3]. It is known that *T. gondii* alters the metabolism of the host cell during the invasion and replication using host-derived nutrients in their own metabolic pathways [4], and that *T. gondii* does not synthesize its own cholesterol but relies mostly on host-derived lipids for their survival [5]. The mechanisms involved in *T. gondii* lipid acquisition are a matter of interest and are still not completely understood. Some studies show the involvement of organelles such as mitochondria and mainly the endoplasmic reticulum (ER) of host cell as suppliers of lipids, thus contributing to the increased area of vacuoles membrane during the development of the parasite [6]. In addition, *T. gondii* infection leads to increased receptor-mediated cholesterol endocytosis by the low-density lipoprotein (LDL) pathway [1,7].

Recent studies have proposed a dynamic role for LD in the host response to intracellular pathogens. Pathogen-induced increased LD formation has been described in bacterial, viral, fungal and parasitic infections where a role for this organelle in intracellular survival and replication of pathogens has been proposed [8,9]. Of note, a close association and/or the presence of host-cell LD in pathogen-containing vesicles has been detected in cells infected with *Mycobacterium tuberculosis*[10,11], *Mycobacterium bovis* BCG [12,13], *Mycobacterium leprae*[14], *Chlamydia*[15], as well as with protozoan parasites *Plasmodium falciparum*[16] and *Trypanosoma cruzi*[17,18], suggesting a role for LD in lipid trafficking during infection.

Structurally, the LD consists of a nucleus of cholesteryl esters and triglycerides surrounded by a single monolayer of phospholipids [19]. The regulated formation of lipid droplets, their protein and lipid content, and their association with other intracellular organelles have established LD as specialized, inducible cytoplasmic domains that function not only in lipid storage but as organelles with roles in cell signaling and activation, regulation of lipid metabolism, membrane trafficking and control of the synthesis and secretion of inflammatory mediators [20,21]. Accordingly, increase of LD numbers produced during infection is related to the generation of eicosanoids, where LDs have been shown as sites of compartmentalization of eicosanoid-forming enzymes and domains involved in the mechanisms of enhanced eicosanoid production during inflammatory and infectious conditions [12,17,22] such as prostaglandin E_2_ (PGE_2_), a product of cyclooxygenase-2 (COX-2) gene induction [23]. However, the LD formation in *T. gondii* and the transference of the host cell lipids to the parasite across the parasitophorous vacuole membrane (PVM) as well as the participation of ER for the maintenance of the intravacuolar parasites were not fully addressed and remain uncertain.

The LDs are also described as sites of storage and synthesis of cytokines. During the past few years SkMC was identified and characterized as a cytokine-producing cell, capable of producing muscle derived cytokines, the myokines, which may participate during infection by intracellular-muscle pathogens such as *T. gondii*[24]. It is known that some cytokines such as interleukin-12 (IL-12), and particularly interferon gamma (IFN-g) are directly involved in cytogenesis [3], the survival rate of *T. gondii* in SkMC [25] and also the integrity of muscle tissue injury [26]. So we studied the formation of LD muscle cells induced by infection with *T. gondii* and investigated if this infection may modulate the production of IL-12 and IFN-g in this cell type. Besides, some researchers have discussed the importance of the host cell type as a determinant for tachyzoite to bradyzoite conversion [27,28]. It has been demonstrated that primary skeletal muscle cells trigger spontaneous *T. gondii* tachyzoite-to-bradyzoite conversion at higher rates than fibroblasts present in these cultures [29,30]. In the past, little attention had been given to the use of SkMC as potential host cells during the study of the toxoplasmosis, despite its well-known participation during the chronic phase of the disease [31], and its role in the route of parasite transmission via consumption of raw or undercooked meat containing Toxoplasma [32]. In the few last years, our group has been working with primary cultures of SkMC as an experimental model for the study of toxoplasmosis *in vitro*, which opens new perspectives in this field [2,27,29,30,33-36]. Since, *T. gondii* diverts a large variety of lipid precursors from host cytoplasm and efficiently manufactures them into complex lipids to its own benefit [4,37,38], we hypothesized a role for LD biogenesis during *T. gondii* infection.

In this study, we have investigated the role of LD biogenesis and their interaction with PV, the modulation of IL-12 and IFN-g secretion as well as COX-2 gene expression and PGE_2_ synthesis, during *T. gondii*-SkMC infection in order to better understand the survival mechanisms of Toxoplasma in muscle cells.

## Methods

All procedures were carried out in accordance with the guidelines established by the Colégio Brasileiro de Experimentação Animal (COBEA), by Fundação Oswaldo Cruz - Fiocruz Committee of Ethics for the Use of Animals (license CEUA LW 10/10) and by the Guidelines on the Care and Use of Animals for Experimental Purposes and Infectious Agents (NACLAR).

### Primary culture of skeletal muscle cells

Skeletal muscle cell primary cultures were obtained from disaggregated thigh muscles of 18-day-old Swiss-Webster albino mouse embryos. The tissues were minced and incubated for 7 min with 0.05% trypsin and 0.01% versene diluted in phosphate-buffered saline pH 7.2 (PBS). After 5–7 dissociation cycles, the enzymatic digestion was interrupted by adding 10% fetal bovine serum at 4°C, the suspension was centrifuged at 650 g for 7 min, resuspended in Dulbecco’s modified Eagle medium (DMEM) supplemented with 10% horse serum, 5% fetal bovine serum, 2% chick embryo extract, 1mM L-glutamine, 1,000 U/ml penicillin, 50 μg/ml streptomycin and then incubated for 30 min at 37°C in a 5% CO_2_ atmosphere. After incubation, the culture flask was gently shaken to release the non-attached cells and the supernatant enriched with myoblasts was seeded in 0.02% gelatin-treated 24-well culture plates (for fluorescent assays) or in 35 mm-culture plates (for electron microscopy studies), respectively. The cultures were maintained at 37°C up to 3–5 days to obtain the muscle fibers and the fresh medium was added every two days. The main characteristics of the cells during myogenesis are: The myoblasts, which are the mononucleated precursor cells of muscle fibers, culture divides two to three times and then begins to aggregate and fuse into postmitotic multinucleated muscle fibers. Moreover, fibroblast phenotype in culture is as follows: the spread exhibited outwards, stellate morphology of fibroblast - like cells and freshly isolated cells demonstrates that the fibroblasts were larger than the myoblasts [36].

### Parasites

Tachyzoites of *T. gondii*, RH strain, were maintained in Swiss mice by serial intraperitoneal inoculation of 10^5^ parasites after 48–72 h inoculation. The parasites were harvested in PBS and centrifuged (200 g for 7–10 min) at room temperature in order to discard blood cells and cellular debris. The supernatant was collected and then centrifuged again at 1000 g for 10 min. The final pellet was resuspended in DMEM and used in the parasite-host cell interaction assays.

### Lipid droplet staining and counting

Muscle cells infected or not with *T. gondii* (parasite: host cell approximate ratio of 5:1) after 6, 24 and 48 h were fixed in 3.7% formaldehyde in HBSS (pH 7.4) and stained with osmium tetroxide, or BODIPY. For the osmium staining, the slides were rinsed in 0.1 M cacodylate buffer, incubated with 1.5% osmium tetroxide (OsO4) for 30 min, rinsed in H_2_O, immersed in 1.0% thiocarbohydrazide for 5 min, rinsed in 0.1 M cacodylate buffer, reincubated in 1.5% OsO4 for 3 min, rinsed in distilled water, and then dried for further analysis. The morphology of fixed cells was observed, and lipid bodies were enumerated by light microscopy with ×100 objective lens in 50 consecutive cells in each slide. The quantitative analysis was based on 3 independent experiments performed in duplicate with at least 200 cells in each coverslip. The person responsible for counting was blinded to the codes for each slide. Slides were alternatively stained with BODIPY, evidencing the accumulation of neutral lipids in lipid droplets. For the BODIPY staining, the slides were fixed in 3.7% formaldehyde for 10 min, washed and incubated for 15 min with the vital stain BODIPY-493/503 (4,4-difluoro-1,3,5,7,8-pentamethyl-4-bora-3a,4a-diaza-s-indacene) or Nile red diluted in PBS, in the proportion of 1:200 and 1:1000 (v/v), respectively. The cultures stained by BODIPY were washed in PBS, incubated for 10 min with 1 μM of To-PRO_3 iodide-642/661 in PBS to enable the visualization of the nuclei of cells, followed by 4 μg/mL phalloidin-TRITC (binds to the actin cytoskeleton) for 1 h at 37°C for better visualization of SkMC. The coverslips were mounted over the sections with 2.5% 1.4-diazabicyclo-(2.2.2)-octane (DABCO). The samples were examined using a Zeiss photomicroscope equipped with epifluorescence and a confocal laser scanning microscope Fluoview 3.2 Olympus, with objective lens of 63× and of 100× (Farmanguinhos/Fiocruz).

The measurement of the area of lipid droplets was done using the BODIPY fluorescent images, obtained with an objective lens of 63× (at least four fields per slide). The images were scanned and analyzed with 2D image software (LSM Image Browser 5). The spots were determined by automatic detection, and the total area of fluorescent lipid bodies were quantified and compared to the lipid bodies of uninfected cells.

### Transmission electron microscopy

SkMC were allowed to interact for 4 to 48 h at 37°C with tachyzoites of *T. gondii* (parasite: host cell approximate ratio of 5:1). After washing in PBS, the uninfected and *T. gondii*-infected SkMC were immediately fixed for 30 min at 4°C in 2.5% glutaraldehyde solution (GA) in 0.1 M Na cacodylate buffer containing 3.5% sucrose and 2.5 mM Ca^+2^, pH 7.2. The cells were then washed in the same buffer and post-fixed for 30 min at 4°C in 1% OsO_4_ in cacodylate buffer. After fixation, the cells were scraped gently from the plastic dish and centrifuged for 5 min at 10,000 g, dehydrated in acetone and embedded in PolyBed 812 resin. Thin sections were contrasted in uranyl acetate and lead citrate and examined in a Zeiss EM10C transmission electron microscope at the Electron Microscopy Platform of Instituto Oswaldo Cruz.

### Ultrastructural cytochemistry for lipid detection

SkMC infected with *T. gondii* for 4 and 24 h were washed in PBS and fixed for 1 h at room temperature with 2.5% GA in 0.1 M Na cacodylate buffer with the addition 3.5% sucrose, pH 7.2. The cultures were washed in the same buffer for 10 min and immediately incubated for another 10 min in 0.1 M of imidazole buffer (CH_3_H_4_N_2_), pH 7.5 [39]. After washing, the cultures were post-fixed for 30 min at room temperature in 2% OsO_4_ diluted in imidazole buffer, pH 7.5, in the dark. After incubation, the cells were washed twice for 10 min in imidazole buffer and then processed for transmission electron microscopy. The ultrathin sections were contrasted with lead citrate for 1 min and examined in a Zeiss EM10C transmission electron microscope.

### Ultrastructural cytochemistry for sarcoplasmatic reticulum (SR) detection

After 4 and 24 h of interaction with *T. gondii*, the infected SkMC cultures were washed in PBS and fixed for 30 min at room temperature in 2.5% GA in 0.1 M cacodylate (pH 7.2). The cells were washed twice for 15 min in the same buffer and washed again twice for 10 min in 1% potassium iodide (KI) diluted in distilled water. The cultures were then incubated for 48 h (in the dark) at room temperature in 1% OsO4 and 1% KI, washed for 10 min in KI solution diluted in distilled water [40] and finally processed as routine for transmission electron microscopy. The ultrathin unstained sections were examined in a Zeiss EM10C transmission electron microscope.

### Cytokine measurement

Cell-free supernatants from muscle cell culture infected by *T. gondii* per 6, 24 and 48 h and controls were harvested and used for IL-12 and INF-g cytokine level measurements by ELISA, following the manufacturer’s recommendations for each kit (Duo Set Kit from R&D systems). The limit of detection of the assay is 40 pg/ml for IL-12 and 30 pg/ml for IFN-g.

### PGE_2_ measurement

PGE_2_ levels were measured directly in the supernatant from uninfected muscle cell cultures and *T. gondii* infected groups after 6, 24 and 48 h of interaction. The PGE_2_ was assayed in the cell-free supernatant by enzyme-linked immunoassay (EIA), according to the manufacturer’s instructions (Cayman Chemical). The limit of detection of the assay is 15 pg/ml.

### Reverse transcriptase polymerase chain reaction (RT-PCR) analysis

Total RNA was extracted from SkMC culture samples harvested at two different time points from experimental *T. gondii* infection assay (after 3 h and 24 h). For this purpose, 10^6^ cells were harvested and washed three times in PBS and centrifuged at 10,000 g. The supernatant was completely removed and the pellet obtained was used for RNA extraction with the RNeasy kit (Qiagen California, CA, USA), according to the manufactor’s recommendations. Total mRNA was measured and cDNA was synthesized using oligo(dT) and Superscript III First-Strand System (Invitrogen,cat#18080-051). Real-time PCR was performed on StepOnePlus using Taqman Gene expression assay: COX-2 (Mm01307334_g1) and HPRT1 (Mm01545399_m1) obtained from Applied Biosystems. Efficiency curve showed between 88–92%. All qRT-PCR experiments were performed in duplicate, including no-template controls. The relative expression of COX-2 was determined using the 2^(-ddCt)^ method.

### Statistical analysis

Data were reported as the mean ± S.E. and were analyzed statistically by means of analysis of variance followed by Student’s *t* test with the level of significance set at *p* ≤ 0.05.

## Results

### *T. gondii* infection triggers lipid droplet biogenesis in muscle cells

**Figure 1:**
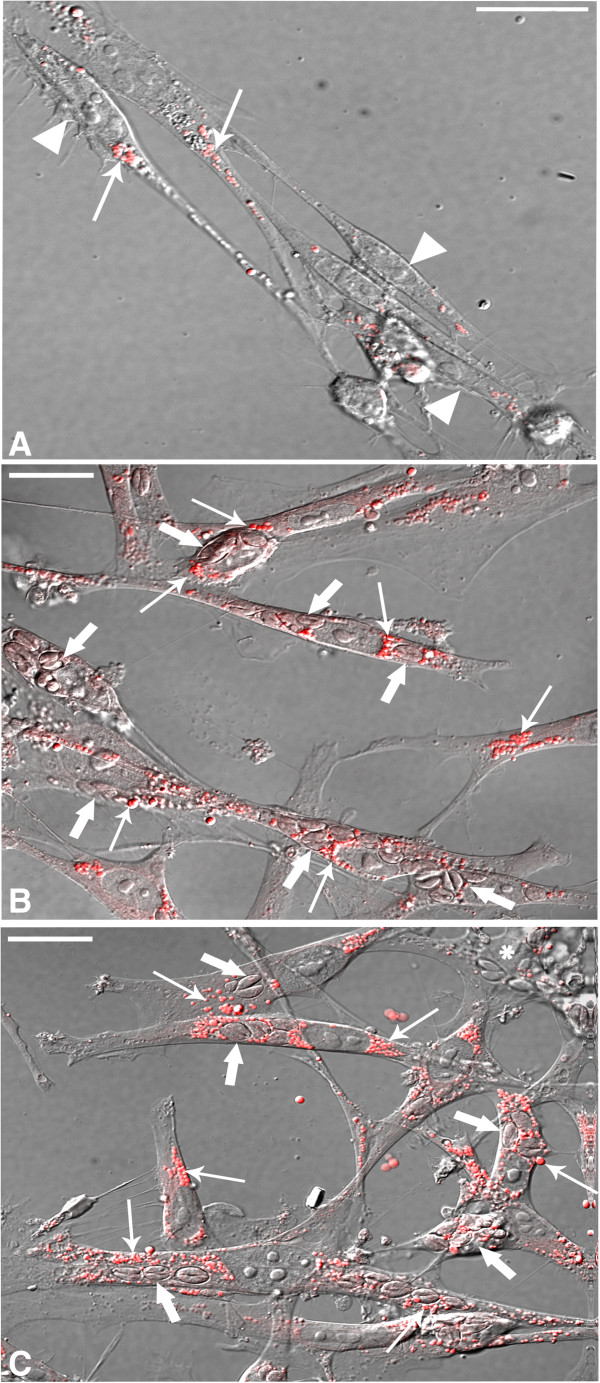
**Confocal laser scanning microscopy showing LD revealed by the Nile Red stain technique in SkMC uninfected and infected with *T. gondii*. (A)** The LD (arrow) in uninfected SkMC (arrowhead) stained in red appears distributed throughout the cell cytoplasm and concentrated around the nucleus (N) (thin arrows). **(B and C)** After 24 h SkMC infection by *T. gondii* a clear association of several LD (thin arrows) can be seen with the PV (thick arrows). Fibroblasts play little LD labeling (*). Bars: A = 50 μm; B and C = 20 μm.

**Figure 2:**
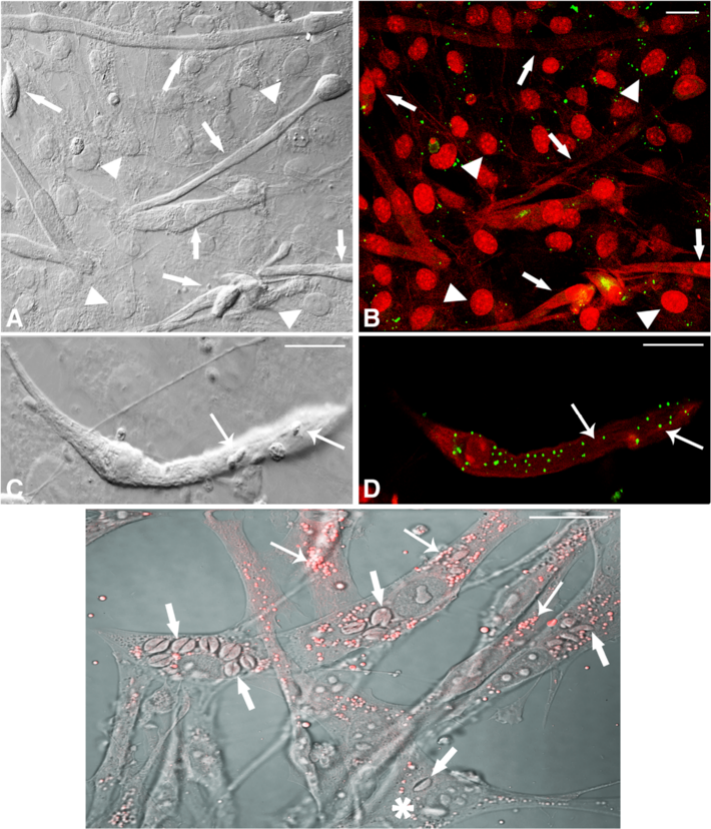
**Confocal Laser Scanning Microscopy showing LD revealed by BODIPY in uninfected and *T. gondii*-infected SkMC for different periods of interaction. (A)** Control - interferential microscopy revealing the profile of uninfected culture with the presence of myoblasts (double arrows), multinucleated myotubes (arrows) and fibroblasts (arrowhead). **(B)** Merge showing double marking for actin cytoskeleton and nuclei SkMC (arrows) in red and LD in green. Note discrete distribution of LD across culture. **(C)** Interferential microscopy showing parasites in *T. gondii*-infected SkMC (thick arrows) after 6 h of the interaction. **(D)** Merge showing actin cytoskeleton and nuclei in red and LD in green in *T. gondii*-infected SkMC. All bars = 20 μm.

**Figure 3:**
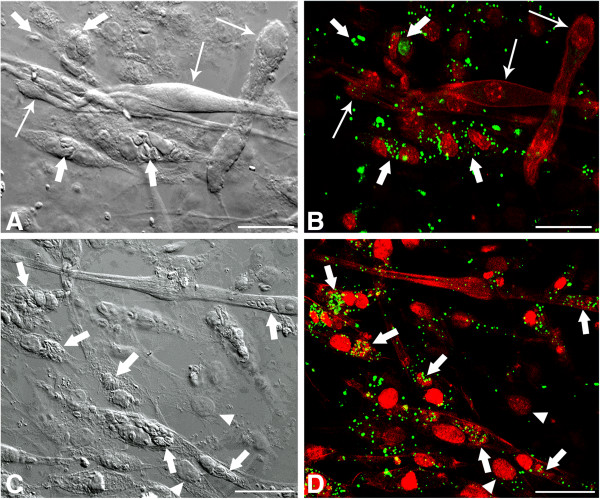
**Confocal Laser Scanning Microscopy analysis showing LD revealed by BODIPY in *T. gondii*-infected SkMC after 24 and 48 h of interaction. (A)** Image by interferential microscopy showing *T. gondii-*infected SkMC after 24 h. Note the presence of infected myotubes (thick arrows) and uninfected (thin arrows) in the same culture. **(B)** Uninfected myotubes (thin arrows) practically does not present LD while cells infected with *T. gondii* (thick arrows) have numerous LD in green. **(C)** Interferential microscopy showing *T. gondii-*infected SkMC after 48 h. Note the presence of infected cells (thick arrows). **(D)** Double staining *T. gondii-*infected SkMC in red and LD in green (arrows). Note the major concentration of LD mainly in infected SkMC. Fibroblasts in culture present few LD infected or not (arrowhead). All bars = 20 μm.

**Figure 4:**
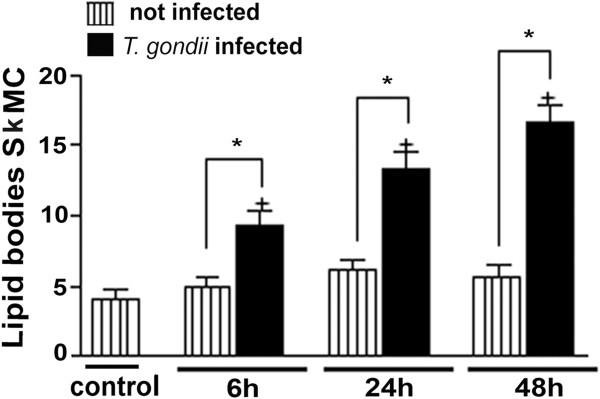
**Biogenesis of LD in SkMC analyzed by osmium stain.** Listed columns represent the profile of LD in SkMC control with preservation synthesis during the development of culture. The black columns represent *T. gondii*-infected SkMC. The time-dependent increase of lipid droplets was observed after 6, 24 and 48 h of *T. gondii*-SkMC interaction. T Test: * P ≤ 0,05.

In order to detect and quantify the incorporation of neutral lipids and LD biogenesis in uninfected and *T. gondii* infected cultures, three different lipid markers, BODIPY 493/503 (4,4-difluoro-1,3,5,7,8-pentamethyl-4-bora-3a,4a-diaza-s-indacene), Nile Red or osmium (OsO_4_) were used. Mixed cultures containing both SkMC and fibroblasts after 24 h and 48 h of cultivation allowed by use of BODIPY a comparative analysis of these cells for the presence of LD. LD revealed by Nile Red stain were distributed throughout the whole cell cytoplasm, sometimes concentrated at the perinuclear region in uninfected SkMC as showed by both interferential and fluorescence microscopy overlay and by confocal microscopy (Figure 1A). After 24 h of infection, many parasites were observed within the PV in SkMC, with a notable presence of LD around these vacuoles (Figure 1B and C). We also demonstrated using BODIPY the slight presence of LD in uninfected cultures in myoblasts, myotubes and also fibroblasts, after 96 h (Figure 2A and B). After 6 h of interaction with *T. gondii* a remarkable increase of LD biogenesis was observed and its recruitment in *T. gondii*-infected SkMC as shown by both interferential (Figure 2C) and confocal microscopy (Figure 2D). However, fibroblasts present in the culture have virtually little or no LD labeling (Figure 1C). Besides, it was possible to demonstrate by use of BODIPY in representative images at 24 h (Figure 3A and B) and 48 h (Figure 3C, D) an increase of LD in infected SkMC, which was time-dependent when analyzed during a period of 6 h to 48 h (Figure 4). Figure 3C and D show that fibroblasts present in culture, whether infected or not, show no changes in the LD numbers. The quantitative analysis of experiments using osmium as stain showed that during myogenesis of SkMC, the number of LD remained constant, but there was a significant time-dependent increase of these structures (about 2–3 times) after 6, 24 and 48 h of interaction with *T. gondii* (Figure 4). At all times of interaction, in addition to increased LD numbers in observed parasitized SkMC, an increase of about 5.2 times in the LD area was also seen as compared to normal cells (p < 0.001), showing the average size of 22.14 μm in uninfected SkMC and about 115.25 μm in infected cells.

### Sarcoplasmic reticulum and LD interact in *T. gondii* infection

**Figure 5:**
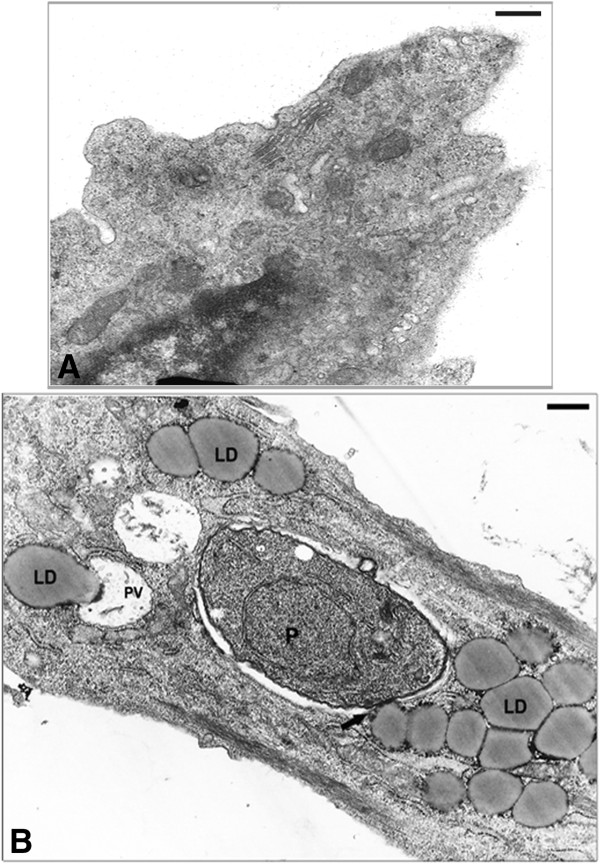
**Ultrastructure of SkMC in cultures. (A)** Uninfected cells without LD. **(B)** Note the large amount of lipid droplets during the first 6 h of *T. gondii*-SkMC interaction. All bars = 10 μm.

**Figure 6:**
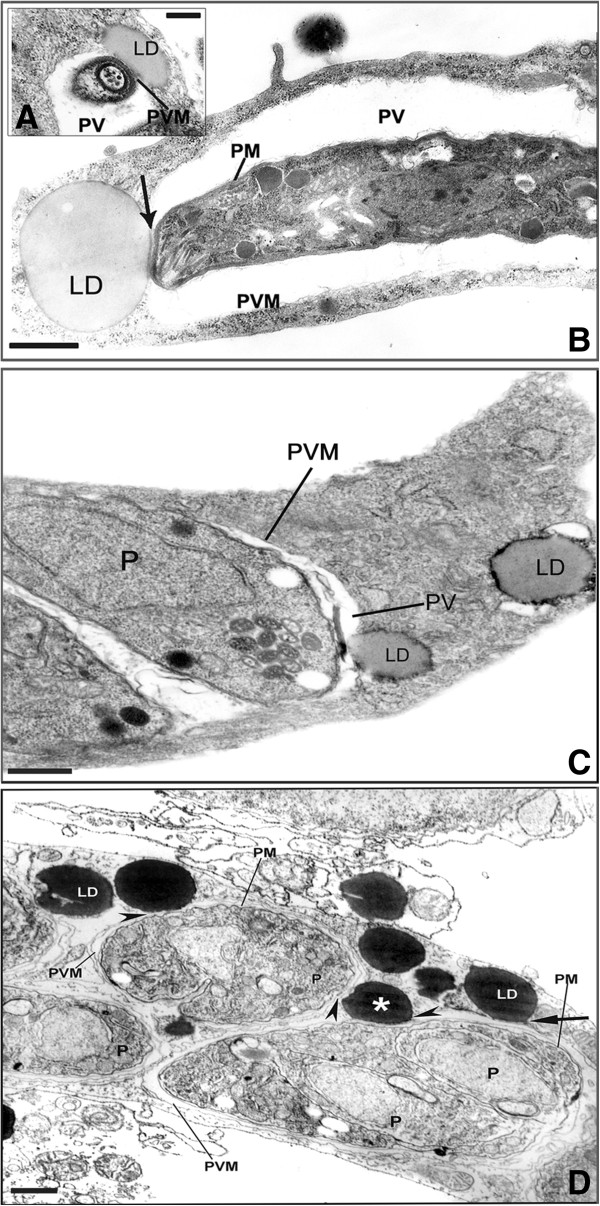
**Ultrastructural analysis of *T. gondii*-SkMC interaction. (A)** The conoid of the parasite (P) was in close contact with LD; **(B and C)** Images showing a large lipid droplets (LD) in closed contact with the parasitophorous vacuole (PV) after 12 h of *T. gondii* - SkMC interaction, respectively. and also vacuolar matrix **(D)** After 24 h SkMC infection by *T. gondii* a clear association of several LD revealed by imidazole can be seen in connection with two PV (asterisks). PM: parasite membrane; PVM: parasitophorous vacuole membrane. Bars: A = 0.2 μm; B-D = 1 μm.

**Figure 7:**
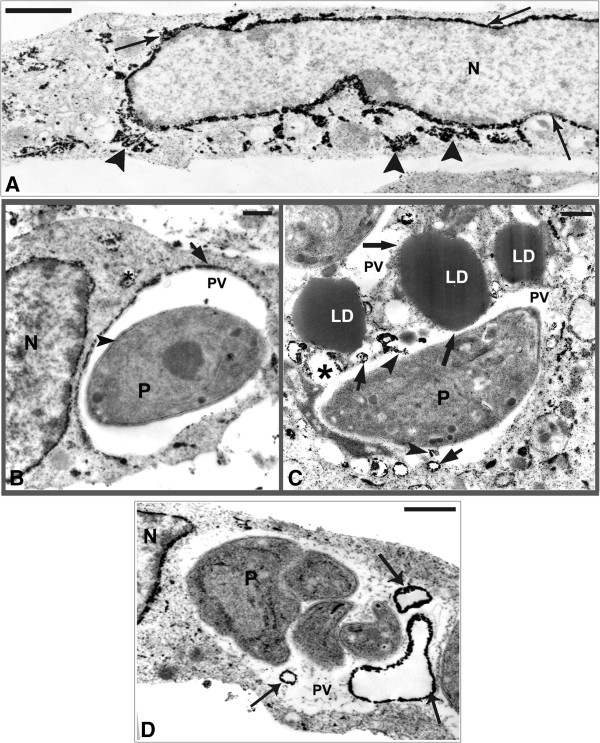
**Ultrastructural cytochemistry using potassium iodide (KI) for detection of sarcoplasmic reticulum (SR). (A)** SR profiles from uninfected SkMC displaying positive reaction products for KI around the nucleus (N) (arrow) and in the cytoplasm (arrowhead). **(B)** SkMC infected for 4 h with *T. gondii* shows the presence of SR profiles containing the reaction product for KI in the cytoplasm (asterisk) surround the nucleus (N) and the PV (arrow). Demarcation in the complex internal membrane of the parasite could also be observed (arrowhead). **(C)** Profiles of SR revealed by KI (small arrow) are observed near and in fusion processes with PV (asterisk) and also inside the PV (arrowhead). Association of several lipid droplets (LD) darkened - revealed by imidazole technique can be seen with the PVM (thick arrow). **(D)** After 24 h of *T. gondii*-SkMC interaction several parasites were observed within the PV containing vesicles of different morphologies and sizes labeled with KI (arrows) and around the nucleus (N). Bars: A = 50 μm; B and C = 0.5 μm; D = 1 μm.

To better understand the results observed by fluorescence analysis we also investigated the involvement of LD by electron microscopy. In this ultrathin section the ultrastructural analysis of uninfected SkMC shows no LD (Figure 5A), while the infected muscle cell after 6 h of interaction displays the VP containing one parasite and the presence of many light dense cytoplasmic structures with a frequent peripheral rim of electron-dense material consistent with LD in the cell cytoplasm, around the VP and in close contact with a typical PVM (Figure 5B). Analysis after 6 h of interaction shows the conoid of the parasite (P) was in close contact with LD (Figure 6A) and also LD were in intimate contact with the PVM and the membrane of the parasite in Figure 6B and also its integration within the matrix of the vacuole (Figure 6C). After 24 h of infection several darkened LD, as revealed by the imidazole technique, could be seen in close contact with PV and some LD simultaneously associated to two different PV (Figure 6D), like a bridge connecting them. The ultrastructural cytochemistry of SkMC using the potassium iodide (KI) technique revealed the presence of tubular structures with the electron dense label distributed over the whole cytoplasm and also in the nuclear envelope (Figure 7A). After 4 h of parasite-SkMC interaction it was noted that the reaction product was localized in structures resembling profiles of sarcoplasmic reticulum (SR), which surrounded the PV, dispersed in the host cell cytoplasm and located around the nucleus, as well as in the inner membrane complex of the parasite (Figure 7B). Precipitation of the reaction product for KI could also be observed inside the PV and in association with the PVM and also LD, as revealed by the imidazole technique (Figure 7C). SkMC after 24 h of parasite-host cell interaction showed cells containing two or more parasites with reaction product for KI surrounding the nucleus of SkMC and in vesicles inside the vacuole containing *T. gondii* (Figure 7D).

### *T. gondii* infection induces cytokine production in skeletal muscle cells

**Figure 8:**
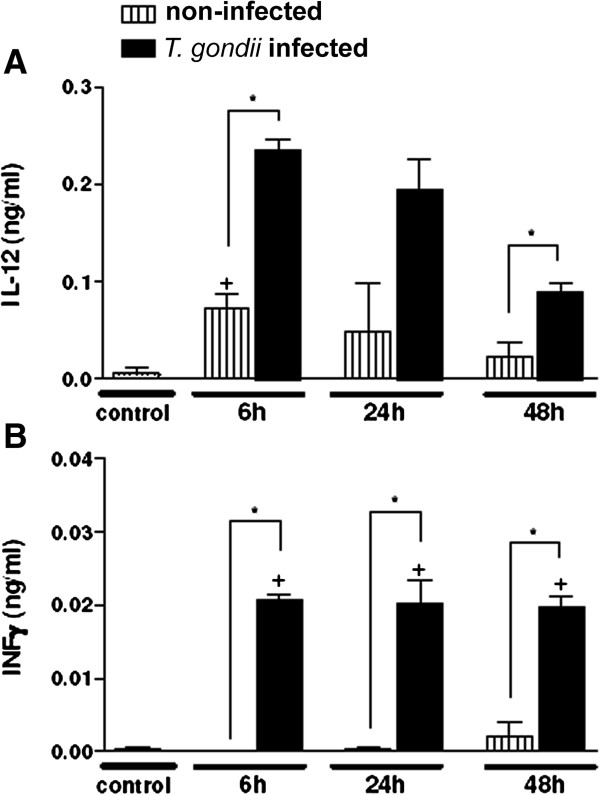
**The graphics show the synthesis of the cytokine interleukin-12 (IL-12) and interferon-g (IFN-g) in *T. gondii-*infected SkMC. (A)** SkMC control represented by the columns listed. Note the decreased synthesis of IL-12 during the development of culture. The dark columns represent infection, showing increased synthesis of IL-12 at all times of interaction. **(B)** Synthesis of the IFN-g in *T. gondii-*SkMC infected after 6, 24 and 48 h. SkMC control represented by the columns listed with a small increase in the synthesis of this cytokine during myogenesis. The dark columns represent infected cultures demonstrating the maintenance of INF-g synthesis at all times of interaction. t-test: *P ≤ 0,05.

In this study we addressed whether SkMC could produce the cytokines IL-12 and INF-g during myogenesis and *T. gondii* infection. SkMC showed a gradual decrease in the synthesis of IL-12, five days after cultivation. However, in *T. gondii* infected-cell cultures, after 6, 24 and 48 h of interaction a significant increase in the production of this cytokine occurred as compared with control. Higher levels of IL-12 were observed in earlier periods of infection analyzed, which were reduced after 48 h of infection (Figure 8A). Increased INF-g levels were verified in the supernatant of *T. gondii*-infected SkMC in all periods analyzed (Figure 8B).

### *T. gondii* infection induces eicosanoid generation in skeletal muscle cells

**Figure 9:**
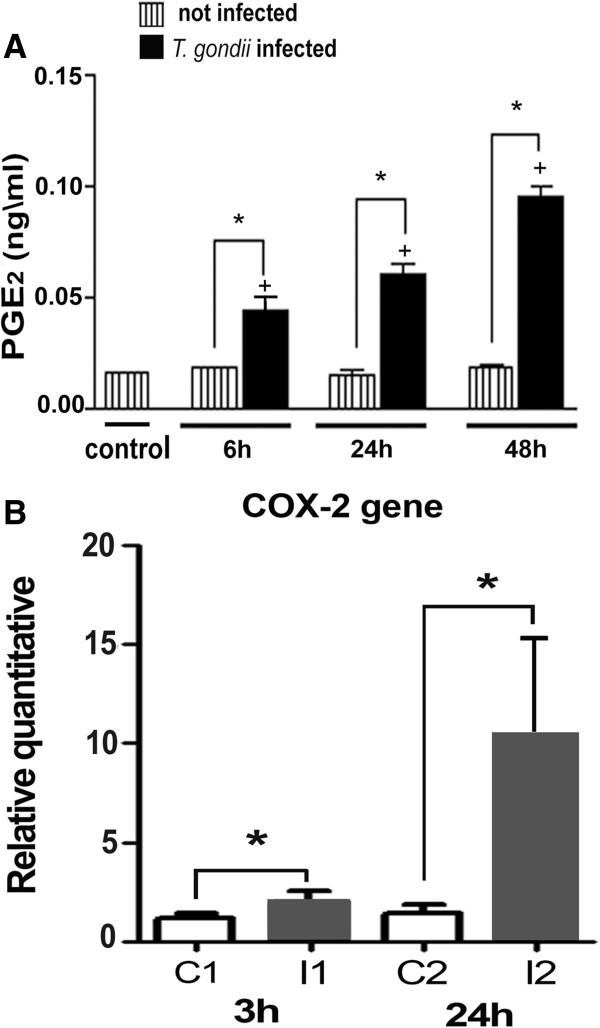
**PGE**_**2 **_**generation and COX-2 gene expression in *T. gondii*-infected SkMC. (A)** Synthesis of PGE_2_ by EIA. The listed columns represent SkMC control with approximate values in all points. The black columns show infected cultures where there is a time-dependent increase in the production of PGE_2_. **(B)** Representation of COX-2 gene expression by RT-PCR in *T. gondii-*infected SkMC after 3 and 24 h. The white columns represent SkMC control with approximate values in all points. The gray columns show infected muscle cells where there is a time-dependent increase in the production of COX-2 gene. t- test: *P ≤ 005.

Lipid droplets are stores of the eicosanoid precursor arachidonic acid in different leukocyte subsets, including eosinophils, neutrophils, and monocytes, and contain eicosanoid-forming enzymes [12,22]. In this way, we investigated whether *T. gondii*-infected SkMC would lead to enhanced PGE_2_ production. PGE_2_ quantified in the supernatant from non-infected and infected muscle cells cultured with *T. gondii* after 6, 24 and 48 h of interaction and measured by enzyme-linked immunoassay, showed a significant time-dependent increase in PGE_2_ generation from 6 h up to 48 h, that parallel and positively correlated with LD formation in *T. gondii*-infected muscle cells but not in uninfected cells (Figure 9A). *T. gondii* infection also triggered a time-dependent increase of COX-2 expression (Figure 9B).

## Discussion

It is known that *T. gondii* mobilizes lipids resources from the host cells during invasion and its intracellular cycle [4], and although the parasite does not synthesize its own cholesterol it has evolved strategies to divert host cell lipid metabolism to favor its survival [1,7]. Our results suggest that *T. gondii*-infected SkMC increases the synthesis of LD as well as their content, providing lipids to the parasite and thus contributing to the growth and maturation of the parasitophorous vacuole, as described in another cellular model by Caffaro *et al.*[38]. Initially using the fluorescent dye Nile Red, it was possible to observe the increased formation of LD within the first hour of *T. gondii-*SkMC interaction and also the presence of LDs next to the parasite, and at 24 h around the PV.

Our quantitative data showed that *T. gondii* infection triggers biogenesis of LD within muscle cells. The analysis by light and fluorescence microscopy of *T. gondii*-infected SkMC stained with osmium tetroxide or BODIPY clearly showed an increase in the number of LD after 6, 24 and 48 h of the interaction. We suggest that Toxoplasma may be interfering with the lipid metabolism of the host cell stimulating its synthesis. Previous studies have shown sequestration of some phospholipids by Toxoplasma infection whilst in the host cell in order to construct more complex lipids [4,41]. In our experiments it was observed that in *T. gondii*-infected SkMC, significant increase in the formation of the LD occurs at all times of interaction: 6, 24 and 48 h. It is known that muscle cells may accumulate phospholipids, triacylglycerol and cholesterol in LD, which could have roles in the regulation of cell cycle, migration and myogenesis by activation of proteolytic systems such as the calpain system [42,43]. Thus, we do not discard the hypothesis that the recruitment of LD by the parasite during its replication, could lead to an increase of LD synthesis by the host cell to maintain homeostasis of their vital activities. The homeostasis is maintained by a balance between the cholesterol internalized via the LDL receptor and synthesis involving the enzyme: 3-hydroxy-3-methylglutaryl-coenzyme A reductase (HMG-CoA) [44]. In cells infected by *T. gondii*, there is an increase in the synthesis of receptors for internalization of LDL [1], and the activity of HMG-CoA is four times higher [45].

Our ultrastructural cytochemistry analysis demonstrated, for the first time, the direct contact of LD with the vacuolar membrane, its matrix as well as with the parasite membrane during its segregation inside the PV. Studies show the involvement of ER from the host cell in the biogenesis of LD [21]. This supplement of lipids could contribute to the increase of the vacuole membrane area during the intracellular development of *T. gondii*[6,38]. LD structure and composition, as determined in different cell types and conditions, consists of cholesteryl esters and triglycerides surrounded by a single monolayer of phospholipids and contains a variable array of proteins [19,21]. We believe that this recruitment of LD by *T. gondii* may be involved in a survival strategy to surmount the deficiency of cholesterol and other lipids by the parasite.

The mechanism by which host-cell-derived lipids are transferred across the PVM to the parasite is uncertain [4]. In addition to LD recruitment, our data clearly demonstrated: (i) the discharge of the SR to the interior of the PV after 4 h of *T. gondii*-SkMC infection; (ii) the presence of vesicles with different diameters and morphology containing the reaction product for KI localized inside the vacuole after 24 h of parasite-host cell infection. These results are similar to that described by Puzianowska-Kuznicka and Kuznicki [46] who observed by immunoelectron microscopy the transfer of SR components into the PV, indicating that the fusion occurs between the two compartments and, (iii) the accentuated decrease of the SR demarcation around the PV, after 24 h of infection. This data suggests that components of the SR can be incorporated by the intracellular parasites, constituting a source of nutrients and lipid possibly for its development, as proposed previously [4,46].

The ER plays a crucial role in cytoplasmic signaling in a variety of cells. It is particularly relevant to SkMC, where this organelle constitutes the main Ca^2+^ store for essential functions, such as contraction [47,48]. Our results by electron microscopy showed total reorganization of the SR in *T. gondii-*infected SkMC. Thus, we believe that this phenomenon may lead to changes in Ca^2+^ homeostasis compromising the functionality of SkMC. The importance of juxtapositioning of SR, mitochondria and transverse tubules (T-tubules) in muscle cell for better communication between sites of Ca^2+^ release which ensures the contraction of myofibrils are described. Therefore, we suggest that *T. gondii* may be benefiting from the repositioning of the SR sequestered, not only lipids, but also from the Ca^2+^ not used by sarcomeres [49].

The accumulation of the LD within leukocytes in inflammatory conditions for example: bacterial sepsis, allergic lung inflammation, arthritis, and in mycobacterium infections among others has recently been reported [21]. However, mechanisms that regulate LD formation and its functional significance to the cellular biology in *T. gondii* infection are not known.

Increased PGE_2_ production in pathological conditions is largely regulated by COX-2 gene induction [23]. By using qRT-PCR the expression of COX-2 gene was analyzed, showing that after 3 and 24 h of *T. gondii*-SkMC interaction, the COX-2 expression was up regulated. Studies have demonstrated that the promoter region of COX-2 has several potential regulatory elements, which can affect gene transcription [50]. In pancreatic beta-cells several transcription factors regulate COX-2 gene expression as for example, the signal transducer and activator of transcription 1 (STAT1) that plays a negative role on COX-2 promoter [23]. It was described that *T. gondii* can also manipulate the host transcription factors, including inhibiting STAT1 [3]. Thus, we suggest that the presence of *T. gondii* in SkMC may be inducing the increased COX-2 expression, but whether or not the inhibition of STAT1 also occurs in this process still remains uncertain.

Several studies have demonstrated that an increase of LD numbers produced during infections by different pathogens is related to the increased generation of eicosanoids due to the compartmentalization of the substrate arachidonic acid and the eicosanoid-forming enzymes within newly formed LDs and LDs are major sites of eicosanoid production in different inflammatory conditions [12,17,18,21,22]. Our results show an increase of PGE_2_ synthesis, from 6 h up to 48 h of *T. gondii-* SkMC infection. Of note, the *T. gondii-*induced increased PGE_2_ generation occurs in parallel and positively correlates to the increased formation of LDs, suggesting that LDs may have roles in the heightened eicosanoid production during *T. gondii* infection. However, future studies will be necessary to confirm the involvement of LDs in the PGE_2_ production triggered by *T. gondii*. Among other factors, we believe that the success of the infection of *T. gondii* in the muscle tissue may be related to the increased COX-2 expression, compartmentalization within LD and consequently enhanced production of the eicosanoid PGE_2_. In other cell types, studies showed that the enzymatic conversion of free arachidonic acid into prostaglandin down-modulates the cell-mediated response favoring not only intracellular pathogens, but also the survival of the host [51,52]. Indeed, high concentrations of PGE_2_ potently inhibit the Th1 type response, tumor necrosis factor (TNF) and nitric oxide (NO) production, and these changes favor intracellular parasite growth [53,54].

It is worth remembering that SkMC is not a cell of the immune system. The levels of inflammatory markers (PGE_2_ and COX-2) observed in SkMC in our assays are considered quantitatively lower when compared to levels produced by macrophages, despite their increase during Toxoplasma infection [52]. We believe that a moderate SkMC immune response may suppress the replication of parasites favoring the bradyzoite conversion, while a strong immune response may change the cell cycle progression parasite or act as a microbicide activator [55]. These data support our hypothesis that the recruitment of LD by *T. gondii*, together with the cellular response, may possibly be related to the development of the chronic phase in SkMC.

Accordingly, intracellular pathogens-induced increased LD formation during intracellular infection, including *T. cruzi*, *M. bovis* BCG and *M. leprae* favouring intracellular pathogen survival through mechanisms which involve LD-derived eicosanoid formation and LD recruitment towards the phagosome [12,14,17]. Similarly, we describe here that the enhanced capacity of muscle cells to generate PGE_2_ in the course of the *T. gondii* infection correlates to the increased LD formation. And so, the recruitment of organelles such as LD and SR by the parasite during its host cell interaction, may contribute to the mechanisms that intracellular pathogens have evolved to survive in host cells. Future studies are necessary to characterize the regulation and function of the prostaglandins in SkMC and to understand if the presence of SR in PV may be acting as a source of Ca^2+^ that facilitates the preference of muscle tissue in the development phase of chronic toxoplasmosis.

In our experiments we observed that SkMC is capable of producing levels of the cytokines IL-12 and IFN-g with a significant increase in their synthesis after 6, 24 and 48 h of interaction with *T. gondii*. Our results corroborate with studies that show the muscle as a cytokine producer by myokines [56]. IL-6 was the first cytokine to be discovered being produced by muscle cells; however, skeletal muscles may produce and express different cytokines families [24,25]. Some authors describe that concerning the synthesis of IFN-g, *T. gondii-*infected SkMC are able to develop a strong anti-parasitic response, reducing significantly the growth of the parasite [25]. Experiments using mice lacking IFN-g [57] and IL-12 showed absence of appropriate immunity, which rapidly leads to host death [58]. It is most advantageous for the Toxoplasma to keep its host alive until transmission to another host through oral transmission of tissue cysts.

IFN-g is known to induce an inflammatory response and control of parasite load during the early stages of infection [59]. During Toxoplasma infection, the host immune response is dependent on IFN-g induced by IL-12 production in a variety of cell types. In our study, in the course of myogenesis of SkMC the levels of IFN-g did not change, whereas the concentration of these cytokines increased in all times of interaction with *T. gondii* analyzed. However, in other cellular models such as macrophages, neutrophils and especially dendritic cells, Lang *et al*. [60] described that *T. gondii* inhibits production of IL-12. Nickdel *et al*. [61] showed that during the early stage of oral infection with *T. gondii* an increase in small-intestine pathology occurred, in addition a reduction in the levels of plasma IL-12 and IFN-g levels was observed. Moreover, Matowicka-Karna *et al*. [62] studying a group of patients infected with *T. gondii*, also noted a decrease in IL-12 levels. We believe that the increase in cytokines IFN-g and IL-12 in SkMC-*T. gondii* infected cells may be related to manipulation of transcription factors of the host by Toxoplasma early on the infection as shown by our results after 3 hours of interaction. Moreover, the increased synthesis of IFN-g [25] and IL-12 may be act by reducing the multiplication process of tachyzoites forms (acute phase) favoring, the differentiation to bradyzoites forms, found in tissue cysts (chronic phase).

Cheng *et al*. [26] have shown that IFN-g expression is upregulated in skeletal muscle following injury. It is of interest to the parasite that the host cell remains alive, thus increasing the synthesis of a cytokine that is involved in the repair and homeostasis of the host cell and may act as a strategy that favors *T. gondii* maintenance. So, Toxoplasma has evolved to exploit own molecules and cellular response of the host, providing a favorable environment for the establishment of chronic infection in SkMC.

Finally, SkMC can be used as an important cellular model for studies on the molecular mechanisms in response to parasitism by *T. gondii*, mainly considering its importance as a target cell for encystment and its role in the transmission of the parasite.

## Conclusions

In conclusion, our data demonstrated that *T. gondii* infection in muscle cells causes a pronounced effect on host-cell lipid metabolism through regulation of LD biogenesis and recruitment of these organelles to PV. The increased LD formation may potentially act as source of prostaglandin production with implications to the host immune response and could represent a source of lipids and other nutrients for parasite survival. Thus, the increase the LD, followed by expression of COX-2 and PGE_2_ in the SkMC may be contributing to the control of the synthesis of IL-12 and IFN-g during infection by *T. gondii*. We believe that the increase of these cytokines involved in the repair, and homeostasis of muscle cells after injury, might contribute to the establishment and maintenance of the chronic phase of *T. gondii* infection in SkMC.

## Competing interests

The authors declare that they have no competing interests.

## Authors’ contributions

HSB conceived, participated in the design and coordination of the study and had the general supervision and complete overview of the project. AFG co-conceived the study, carried out most of the experimental work, including the processing of samples and the final illustrations for the manuscript, analyzed data and drafted the manuscript, as part of her PhD thesis. RM participated in the electron microscopy assays. LC participated in the design of the study. RMR carried out the molecular assays. KGM and PTB made substantial contributions to data acquisition, analysis and participated in the revision of the manuscript. All authors analyzed the data and read and approved the final version of the manuscript.
